# Research progress in cardiotoxicity of organophosphate esters

**DOI:** 10.3389/fphar.2023.1264515

**Published:** 2023-11-21

**Authors:** Hui Fang, Longfei Yang, Meng Yan, Yanan Fan, Jianping Zhu, Lizhen Ji

**Affiliations:** College of Life Sciences, Shandong Normal University, Jinan, China

**Keywords:** organophosphate esters, cardiotoxicity, pericardial edema, heart rate, signal transduction

## Abstract

Organophosphate esters (OPEs) have been extensively utilized worldwide as a substitution for brominated flame retardants. With an increased awareness of the need for environmental protection, the potential health risks and ecological hazards of OPEs have attracted widespread attention. As the dynamic organ of the circulatory system, the heart plays a significant role in maintaining normal life activities. Currently, there is a lack of systematic appraisal of the cardiotoxicity of OPEs. This article summarized the effects of OPEs on the morphological structure and physiological functions of the heart. It is found that these chemicals can lead to pericardial edema, abnormal looping, and thinning of atrioventricular walls in the heart, accompanied by alterations in heart rate, with toxic effects varying by the OPE type. These effects are primarily associated with the activation of endoplasmic reticulum stress response, the perturbation of cytoplasmic and intranuclear signal transduction pathways in cardiomyocytes. This paper provides a theoretical basis for further understanding of the toxic effects of OPEs and contributes to environmental protection and OPEs’ ecological risk assessment.

## 1 Introduction

Organophosphate esters (OPEs) are a class of phosphate derivatives containing organic groups or compounds containing carbon-phosphorus bonds, with a common skeleton structure of phosphate esters. According to the different functional groups of the side chains, they can be roughly divided into three types: chlorinated OPEs, alkyl OPEs, and aryl OPEs (Tian et al., 2023). Chlorinated OPEs are highly hydrophilic and volatile, and can migrate to environmental media through various physical and chemical processes, with strong hydrolysis and biodegradation resistance (Gao et al., 2015). The common types of chlorinated OPEs include tris (2-chloroethyl) phosphate (TCEP), tris (3-chloropropyl) phosphate (TCPP), tris (1,3-dichloro-2-propyl) phosphate (TDCPP), etc. Alkyl OPEs have a wide range of polarity and significant differences in physical and chemical properties, with common types such as tris (2-butoxyethyl) phosphate (TBOEP) and tributyl phosphate (TnBP). Aryl OPEs have strong hydrophobicity and are prone to bioconcentration, with common types being triphenyl phosphate (TPhP), cresyl diphenyl phosphate (CDP) and so on (Gu et al., 2023).

At present, OPEs have been used widely as flame retardants and plasticizers worldwide, often added to industrial products such as electronic devices, building materials, textiles, and plastics, playing a role in resisting the spread of flames as well as increasing the plasticity and flexibility of polymers (Zhang et al., 2018; Liao et al., 2022). However, as physical additives, OPEs are prone to be released into the environment during production, transportation, utilization, and recycling processes through diffusion, abrasion, and other means (Bacaloni et al., 2007; Sundkvist et al., 2010). Currently, they have been widely detected in various environmental media such as the atmosphere, soil, sediment, water bodies, and even in organisms, human blood and urine.

In recent years, the issue of environmental pollution caused by OPEs has gradually gained the attention of researchers. Numerous studies have confirmed that OPEs can induce various toxicities in organisms, including developmental, reproductive, neurological, metabolic, and endocrine disruptions. For instance, after exposure to TDCPP with environmental concentrations of 300 ng/L and 3,000 ng/L, the growth ability, survival rate, and reproductive ability of *Daphnia magna* were significantly inhibited after 32–90 d depending on the concentration (Li et al., 2017). Tricresyl phosphate (TCP) at 20 μg/L disrupted the balance between excitation and inhibition in the neural circuit of zebrafish (*Danio rerio*), specifically inducing hyperactivity and seizures, which in turn led to severe neurotoxicity (Knoll-Gellida et al., 2021). Impaired liver metabolism and imbalanced gill ion transport were observed in juvenile medaka fish (*Oryzias latipes*) after exposure to TCEP with a concentration of 1 μg/L for 30 d, and cell apoptosis was induced through the p53-Bax pathway and caspase-dependent pathways at 10 μg/L (Zhao et al., 2021). The TPhP at 40 μg/L affected hormone synthesis and thus cause endocrine disruption by damaging the nervous system’s normal regulatory process of thyroid hormone secretion in zebrafish (Kim et al., 2015).

The heart, as the dynamic core of blood circulation in an organism, plays a crucial role in maintaining regular life functions. The investigation of cardiotoxicity holds great significance in drug development and the evaluation of environmental pollution levels. It has become an important focal point in the realms of life sciences, ecotoxicology, and environmental science. It has been confirmed that biological exposure to environmental pollutants can induce cardiotoxicity, which manifests as myocardial injury, disruption of cardiac electrophysiological characteristics, decline in heart function, and the inability to supply sufficient blood to the body, ultimately resulting in myocardial disease (Cross et al., 2015; Zakaria et al., 2018). This paper provides a concise summary of the cardiotoxicity of OPEs, including alkyl, aryl and chlorinated OPEs. Furthermore, it delves into the underlying toxic mechanisms associated with these chemicals, aiming to offer valuable reference materials for toxicological research and ecological risk assessment of OPEs.

## 2 Cardiotoxicity of organophosphate esters

Compared to other organ toxicities, cardiotoxicity is often manifested as myocardial lesions and a decline in cardiac function, characterized by a long latency period, slow progression, and irreversible consequences once established. This section summarizes the cardiotoxicity induced by OPEs, considering morphological structure, physiological function, as well as key molecules and biomarkers.

### 2.1 Effects of OPEs on cardiac morphological structure

In current studies, the main focus is on the cardiac developmental toxicity of zebrafish. This organism has been used widely in exploring cardiotoxicity due to the high similarity of their cardiac developmental process and the high homology of their genome with mammals.

When exposed to nine OPEs at different concentration gradients, zebrafish embryos generally showed pericardial edema and abnormal cardiac looping, manifested by prolonged venous sinus-arteriolar bulb (SV-BA) distance and dioxin-like tubular heart. The above phenomena were positively correlated with the exposure concentration (Du et al., 2015; McGee et al., 2013). Further studies revealed that embryos exposed to TPhP and CDP were more prone to significant pericardial edema. The 96 h-EC_50_ values for these two groups were lower than half of the 96 h-LC_50_ value, whereas the other OPEs groups exhibited values higher than the 96 h-LC_50_. This suggests that aryl OPEs exhibit stronger toxicity to cardiac development. Wiegand et al. (2022) exposed zebrafish embryos to TPhP and observed cardiac looping abnormalities and pericardial edema, occurring within the sensitive time window of 24–30 hpf. Previous studies have confirmed that ionocytes express the ion transporters to maintain ion balance between the aquatic environment and the embryo environment before zebrafish gill development (Guh et al., 2015). It is hypothesized that the formation of pericardial edema may be associated with an increased abundance of ionocytes rich in Na^+^/K^+^ ATPase, known as NaRCs. Further investigations have revealed that the pericardial edema induced by TPhP in zebrafish embryos is dependent on the ion strength of the exposure medium. Therefore, it is of vital importance to further standardize the exposure culture medium and the embryo rearing protocols in zebrafish-based chemical toxicity screening assays (Wiegand et al., 2023).

Furthermore, observation of tissue slices revealed that exposure to 0.50 or 1.0 mg/L of TPhP, or 0.10, 0.50, or 1.0 mg/L of CDP, led to a reduction in the number of myocardial cells and thinning of the atrioventricular wall in zebrafish. This suggests that both TPhP and CDP can affect cardiac development during zebrafish embryogenesis, and CDP exhibits stronger cardiotoxicity than TPhP at the same concentrations (Du et al., 2015). A study by Xiong et al. (2022) also found that continuous intragastric administered of 10 mg TCEP/kg b.w./d for 30 d caused inflammatory infiltration and nuclear swelling or atrophy in mouse (*Mus musculus*) cardiomyocytes. Additionally, it induced myocardial fibrosis, leading to disorganized arrangement of muscle fibers and the appearance of myocardial congestion phenotypes. Further observation of cardiac ultrastructure revealed that exposure to TCEP can induce the formation of mitophagosomes and an increase in the quantity of autophagic vacuoles in myocardial cells in mice. These findings suggest a relationship between TCEP-induced myocardial fibrosis and autophagy. Kanda et al. (2021) investigated the effects of TCEP on chicken embryos and found that the heart weight-to-body weight ratio significantly increased in the group exposed to 500 nmol TCEP/g egg. Additionally, after 3 d of treatment, both the total length of blood vessels and the number of branches showed a significant decrease, suggesting that TCEP induced myocardial hypertrophy and inhibited angiogenesis in chicken embryos.

### 2.2 Effects of OPEs on cardiac physiological function

The heart, being the most essential organ in vertebrate bodies, plays a vital role in propelling blood circulation throughout all body parts. Hence, maintaining proper cardiac physiology is imperative for sustaining normal physiological activities of organisms. Studies have revealed that TPhP, TnBP, and TBOEP can elicit a concentration-dependence decrease in heart rate in Japanese medaka and zebrafish embryos (Sun et al., 2016; Liu et al., 2017). Alzualde et al. (2018) utilized zebrafish embryos to investigate the cardiotoxicity of TPhP, isopropylated phenyl phosphate (IPP), 2-ethylhexyl diphenyl phosphate (EHDP), tert-butylated phenyl diphenyl phosphate (BPDP), trimethyl phenyl phosphate (TMPP), isodecyl diphenyl phosphate (IDDP), and TDCPP. The results revealed that all seven OPEs induced bradycardia in zebrafish embryos. In addition, four non-halogenated OPEs (BPDP, IPP, TMPP, TPhP) showed significant cardiotoxicity at concentrations of 10–100 μM, which was characterized by bradycardia initially, followed by atrial standstill at higher concentrations. Du et al. (2015) investigated the sensitive period of heart development in zebrafish. Zebrafish embryos ranging from 0 hpf to 60 hpf were exposed to CDP or TPhP for 12 h. At 72 hpf, the heart rate and SV-BA distance were measured. The results revealed that zebrafish larvae exposed to 0.5 mg/L TPhP or 0.1 mg/L CDP (the former is about 1/3 of 96h-LC_50_, while the latter is close to 1/10) exhibited an irreversible decrease in heart rate and an elongated SV-BA distance between 0–48 hpf, resembling the dioxin-like tubular heart phenotype. This suggests that these two OPEs can induce bradycardia and inhibit cardiac looping, leading to abnormalities in cardiac circulation (SV-BA distance reflects changes in the position of the atrium and ventricle, serving as an indicator for assessing cardiac circulatory function). Importantly, bradycardia induced by toxicant exposure after 48 hpf could be restored to normal levels after removal of the toxicant for 12 h. The results suggest that zebrafish embryos exhibit a sensitive window for heart development between 0–48 hpf, during which they are more susceptible to pharmacological stimulation. Additionally, in experiments involving exposure of chicken embryos to TCEP, it was found that the heart rate decreased in a concentration-dependent manner and significantly decreased after 4 d of toxicant treatment (Kanda et al., 2021).

Researchers have also conducted *in vitro* experiments to investigated the effects of OPEs on the pulsation of myocardial cells and the differentiation of stem cells into myocardial cells. Sirenko et al. (2017) employed an organotypic human induced pluripotent stem cell-derived model to investigate the *in vitro* cardiotoxicity study on seven OPEs, including EHDP, phenol, isopropylated, phosphate (3:1) (PIP 3:1), TCEP, TPhP, IDDP, BPDP, and TCP. They evaluated the beating behavior of cardiomyocytes by assessing the parameters such as peak frequency, rise time, and decay time of intracellular Ca^2+^ flux at two time points, specifically 30 min and 24 h post-exposure. It was observed that besides TCEP, six other OPEs exhibited a non-monotonic concentration response in myocardial cells, characterized by an increase in peak frequency at low exposure concentrations followed by suppression at higher concentrations. Furthermore, prolonged exposure for 24 h resulted in similar changes with IDDP, BPDP, and PIP 3:1; whereas EHDP and TPhP primarily demonstrated inhibitory effects at high concentrations. In a separate *in vitro* experiment conducted by Qi et al. (2019), it was demonstrated that TPhP significantly reduced the beating frequency of embryoid bodies that formed during the cardiomyogenic differentiation of mouse embryonic stem cells (mESCs), indicating that TPhP can reduce the differentiation of mESCs into cardiomyocytes.

By employing Hoechst 33342 and the mitochondria-specific dye JC-10 co-stain technique, researchers discovered that OPEs could lead to a reduction in the quantity of granules per cell, average granule area, and/or granule intensity within myocardial cells, while also disrupting mitochondrial membrane potential (Sirenko et al., 2017). Transmission electron microscopy examination of mitochondrial structure further revealed that exposure to TCEP induced abnormal increases in small granular mitochondria as well as mitochondrial swelling accompanied by degeneration or disappearance of cristae within mouse myocardial cells (Xiong et al., 2022). These findings collectively suggest that OPE exposure can influence mitochondrial metabolism within myocardial cells.

### 2.3 Effects of OPEs on key molecules and biomarkers in the heart

The morphogenesis and functional maintenance of the heart require the involvement of genes, proteins, and enzymes in a finely regulated way. Researchers utilized zebrafish and mice as experimental models to investigate the effects of OPEs on key genes involved in bone morphogenesis protein 4 gene (*bmp4*) and biomarkers including creatine kinase (CK), among others.

The bone morphogenetic protein BMP4 plays a crucial role in the development of the cardiac outflow tract (OFT), and its functional loss can lead to embryonic lethality and defects in myocardial differentiation in mice (Zheng et al., 2021). In addition, reports have indicated that BMP4 can regulate heart looping and asymmetric development in zebrafish (Chen et al., 1997). The *nkx2.5* belongs to the NK homeobox family and plays a critical role in cardiac cell proliferation and differentiation, ventricular chamber formation, and the development and maintenance of the specialized conduction system; it is a key transcription factor in cardiac development (de Sena-Tomás et al., 2022). The *gata4* can be expressed as GATA binding protein 4, which can activate the αT-catenin promoter in cardiomyocytes, promoting the expression of αT-catenin; *gata4* plays a crucial role in the assembly of the cytoskeleton and myofiber formation in cardiac cells, and is important in local muscle regeneration and cell proliferation during the process of regeneration (Vanpoucke et al., 2004). The *tbx5* plays a crucial role in the formation of the atrioventricular septum, generation of the conduction system, and rhythm control during cardiac development. In addition, the *tbx5* also participates in the process of cardiac regeneration, and its loss can lead to failed heart looping and heart failure in zebrafish (Steimle and Moskowitz, 2017). Du et al. (2015) exposed zebrafish to 0.5 mg/L TPhP and CDP and examined the expression of cardiac developmental regulatory genes *gata4*, *bmp4*, *nkx2.5*, and *tbx5*. During the initial stages of development, the expression of *bmp4*, *nkx2.5*, and *tbx5* were downregulated. Further morphological and functional indicators revealed that zebrafish exhibited decreased heart rate and abnormal development of the specialized cardiac conduction system during the 0–24 hpf stage. As development progressed, the expression of *gata4*, *nkx2.5*, and *tbx5* gradually increased, reaching levels close to, or exceeding those of, the control group by 72 hpf. However, the expression of *bmp4* remained lower than that of the control group throughout, indicating that TPhP and CDP affect cardiac looping and asymmetric development processes. The *Hox* gene family plays a crucial role in regulating the fate of cardiac cells within the second heart field (SHF), contributing to embryonic heart field positioning and establishing the anterior-posterior polarity during heart morphogenesis (Lo and Frasch, 2003; Waxman et al., 2008). Previous research has demonstrated that exposing zebrafish embryos at 6 hpf to 0.2 μM monosubstituted isopropylated triaryl phosphate (mITP) until 48 hpf leads to downregulation of transcription within the *Hox* gene family, resulting in cardiac malformations (Haggard et al., 2017).

The CK catalyzes myocardial cell metabolism and regulates cardiac electrophysiological activity; its abnormal elevation is often considered one of the diagnostic markers for myocardial infarction (Amani et al., 2013). Xiong et al. (2022) found increased protein levels of collagen I, collagen III, and *α*-SMA in mice after exposure to TCEP, accompanied by elevated levels of CK and creatine kinase isozymes (CK-MB), indicating the presence of myocardial fibrosis and impaired cardiac function. Telethonin (TCAP), encoded by the titin-cap gene, is a critical molecule for myocardial sarcomere assembly and regulation. Variations in TCAP can lead to myocardial hypertrophy and an increased risk for dilated cardiomyopathy (Hayashi et al., 2004; Webber et al., 2012). Mitchell et al. (2018) observed that exposure of zebrafish at 72 hpf to TPhP concentrations of 5, 10, and 20 μM resulted in arrested cardiac development and a significant increase in TCAP expression within the heart.

In summary, OPEs can induce cardiac structural changes characterized by pericardial edema, physiological functional changes manifested as abnormal heart rate, and alterations in the expression levels of key molecules and biomarkers such as *bmp4* and CK. The manifestations of cardiotoxicity, changes in key molecules or biomarkers, exposure concentrations, and environmentally relevant concentrations resulting from major OPEs are shown in Table 1.

**TABLE 1:** The cardiotoxic manifestations, exposure concentrations, key molecules or biomarkers of different OPEs.

OPEs	Experimental subjects	Cardiotoxic manifestations	Key molecules and biomarkers	Experimental exposure concentrations	Environmentally relevant concentrations	References
CDP	Zebrafish (*Danio rerio*)	pericardial edema	*bmp4*	0.1–1.0 mg/L	N.D. - 42.2 ng/L	Du et al. (2015), Xing et al. (2023)
CAS: 26444-49-5		decreased heart rate	*nkx2.5*			
		prolonged SV-BA distance	*gata4*			
		decreased cardiomyocytes	*tbx5*			
		thinning of atrioventricular walls				
TBOEP	Zebrafish (*Danio rerio*)	pericardial edema	*nkx2.5*	200–2000 μg/L	4.2–10186 ng/L	Liao et al. (2022), Xion et al. (2021)
CAS: 78-51-3		decreased heart rate	*gata4*			
		increased cardiomyocyte apoptosis	*tbx5*			
		increased oxidative stress				
TCEP	Mouse (*Mus musculus*)	myocardial fibrosis	CK and CK-MB	10 mg/kg b.w	7–1197 ng/L	Liao et al. (2022), Xiong et al. (2022)
CAS: 115-96-8		disarrangement of myocardial fibers	collagen I			
		myocardial congestion	collagen III			
		nuclear swelling or atrophy	α-SMA			
		inflammatory infiltration				
		Ca^2+^ overload				
		ER stress				
		excessive autophagy				
		shrinking and rounding of mitochondria				
		degeneration or disappearance of mitochondrial cristae				
	Chicken embryos	myocardial hypertrophy		50–500 nmol/g		Kanda et al. (2021)
		decreased heart rate				
		delayed development of the cardiovascular system				
TPhP	Zebrafish (*Danio rerio*)	pericardial edema	*bmp4*	0.1–6.5 mg/L	0.07–360 ng/L	Liao et al. (2022), Du et al. (2015), Mitchell et al. (2018), Guo et al. (2017)
CAS: 115-86-6		decreased heart rate	*nkx2.5*			
		prolonged SV-BA distance	*gata4*			
		decreased cardiomyocytes	*tbx5*			
		thinning of atrioventricular walls	TCAP			
	Human iPSC-derived cardiomyocytes	reduced mitochondrial membrane potential	—	3–30 μM		Sirenko et al. (2017)
		decreased mitochondria				
	Mouse (*Mus musculus*)	reduced differentiation capacity of cardiomyocyte	—	38.35–200 μM		Qi et al. (2019)
EHDP	Human iPSC-derived cardiomyocytes	Ca^2+^ overload	—	3–30 μM	0–5400 ng/L	Sirenko et al. (2017), Loos et al. (2013)
		tachycardia (positive inotropic effect)				
CAS: 1241-94-7		reduced mitochondrial membrane potential				
		decreased mitochondria				

N.D.: not detected; ‘-’: no data. The italic values in the second column are the Latin name of the experimental subjects, and the ones in the fourth column are related genes.

## 3 Mechanisms associated with cardiotoxicity caused by OPEs

### 3.1 Wnt signaling pathway

The Wnt signaling pathway is involved in regulating several processes in animal growth and development. It consists of two pathways: the *ß*-catenin-mediated signaling pathway activated by the wnt ligands binding to Frizzled receptors on the plasma membrane; and the non-canonical signaling pathway that involves protein kinase C and Ca^2+^, which is not dependent on the *ß*-catenin-mediated signaling pathway. Excessive activation or inhibition of the Wnt signaling pathway can have significant effects on the development of vertebrate organisms.

The Wnt signaling is essential for the development of the vertebrate heart. The researchers artificially activated the Wnt/*β*-catenin signaling at different developmental stages in zebrafish embryos and found that at 0–5 hpf, it upregulated the expression of *nkx2.5*, a key transcription factor for heart development, and promoted heart development, whereas at 6-9 hpf, it downregulated the *nkx2.5* expression, ultimately inhibited cardiac formation. These findings suggested that the Wnt signaling is involved in early-stage cardiac development by promoting differentiation but suppresses it later on (Ueno et al., 2007). Xiong et al. (2021) observed that acute exposure of zebrafish embryos to TBOEP resulted in the inhibition of both Wnt classical and non-classical signaling pathways. In addition, with the exposure concentration above 1,000 μg/L, the *β-catenin*, *wnt11* and *pkc* were downregulated, and the negative regulators *axin1* and *axin2* were upregulated, ultimately leading to downregulation of the downstream target genes *sox9b* and *nkx2.5*, resulting in abnormal cardiac development. In addition, the disruption and dysregulation of Wnt signaling can induce apoptosis and oxidative stress (He et al., 2005; Lu et al., 2011; Zhang et al., 2013). Under exposure to TBOEP at 2000 μg/L, the number of apoptotic cells and the content of reactive oxygen species in the zebrafish heart region increased, and the activities of superoxide dismutase and catalase decreased (Xiong et al., 2021). The researchers further applied the Wnt signal activator 6-bromoindirubin-3′-oxime (6BIO) to verify the toxic effect of TBOEP on the heart. The results showed that the combined exposure of TBOEP and 6BIO significantly inhibited the upregulation of *axin1* and *axin2*, as well as the downregulation of *β-catenin*, *wnt11* and *pkc*, indicating that 6BIO can alleviate the toxic effect of TBOEP. Small molecule modulators are an important research direction for developing targeted drugs, and the above research provides ideas for exploring intervention measures regarding the toxic effects of OPEs. Up to now, no report has been found on the cardiotoxic effects of other alkyl OPEs or two other types of OPEs through the Wnt signaling pathway. Further research is needed to determine whether they can also interfere with the Wnt signaling regulation.

### 3.2 Calcium overload/endoplasmic reticulum stress/autophagy pathway

The Ca^2+^ ion plays a crucial role in maintaining normal physiological function of the heart, with an imbalance in calcium homeostasis leading to cardiac diseases such as myocardial infarction, arrhythmias, myocardial hypertrophy, and heart failure (Morciano et al., 2022). Endoplasmic reticulum (ER) stress is an adaptive response of cells to the accumulation of proteins in the ER. Excessive stress can lead to calcium overload and oxidative stress, both of which can cause mitochondrial dysfunction, resulting in a reduction in the activity of mitochondrial complex I in the cardiac muscle (Mohsin et al., 2020). Sarco/ER Ca^2+^ ATPase (SERCA) is essential for the removal of excess Ca^2+^, ATP synthesis, and maintenance of normal cardiac function, and is also a target for the toxic effects caused by acute exposure to brominated flame retardants (Al-Mousa and Michelangeli, 2014; Shareef et al., 2014). In mouse cardiomyocytes, SERCA2a is the main subtype whose dysfunction leads to ischemic heart disease and dilated cardiomyopathy. Deletion of the *SERCA2a* gene results in sustained Ca^2+^ influx, causing abnormal cell death and promoting the occurrence of cardiovascular diseases (Chemaly et al., 2018). After 30 d of oral gavage treatment with TCEP at a dose of 10 mg/kg b.w./d, the mice exhibited decreased SERCA expression, accompanied by a significant increase in Ca^2+^ concentration and reduced ER transmembrane protein expression. Furthermore, the Phosphatidylinositol 3 kinase-mechanistic target of rapamycin-Protein kinase B (PI3K/AKT/mTOR) signaling pathway was inhibited. This inhibition resulted in an increased number of autophagic vacuoles and mitochondrial autophagosomes in myocardial cells, indicating that TCEP may induce calcium overload, ER stress, and excessive autophagy in cardiac myocytes by suppressing SERCA expression, ultimately contributing to myocardial fibrosis and cardiac toxicity (Xiong et al., 2022).

In this study, researchers also used a small molecule regulator to verify the cardiotoxicity of TCEP. The SERCA activator CDN1163 has been confirmed to be able to treat diabetes and liver metabolic dysfunction in mice by improving Ca^2+^ homeostasis (Kang et al., 2016). When TCEP and CDN1163 were applied together, a significant improvement in myocardial fibrosis induced by TCEP was observed. It is speculated that CDN1163 suppressed calcium overload by restoring the function of SERCA, reorganized mitochondrial structure, promoted ATP production, and thus alleviated myocardial fibrosis.

### 3.3 Nuclear receptor pathway

Nuclear receptors, which are ligand-dependent transcription factors and belong to a family of proteins, exert critical regulatory roles in various physiological processes such as development, metabolism, reproduction, inflammation, and circadian rhythms. By binding to homologous ligands or specific DNA sequences, they activate or inhibit the transcription of target genes. Dysregulation of nuclear receptor function can contribute to cardiovascular diseases, malignant tumours, metabolic disorders, and inflammatory diseases (Weikum et al., 2018). At present, nuclear receptors are primarily classified into three groups: steroid receptors (Class I receptors), non-steroid receptors (Class II receptors), and orphan receptors (referring to nuclear receptors whose endogenous ligands have not been identified) (Kurakula et al., 2014). The retinoic acid receptors (RARs), belonging to Class II receptors, are essential in the process of heart development. They are instrumental in cardiac morphogenesis, myocardial growth, and coronary artery formation. Mutations in RARs can lead to myocardial thinning and embryonic lethality (Merki et al., 2005). The absence of retinoic acid (RA) during the 4-hpf blastocyst stage can lead to an excessive number of zebrafish myocardial cells, causing an expansion of the SHF after the formation of the heart canal, ultimately leading to the disintegration of the heart canal (Ryckebusch et al., 2008). Upon activation by ligands, the RARs undergo heterodimerization with other Class II nuclear receptors, Retinoid X receptors (RXRs). They bind to the RA response elements, thereby driving the transcription of genes associated with heart development (Minucci et al., 1997). The peroxisome proliferator-activated receptor gamma (PPARγ), also a member of Class II receptors, can be activated by the same ligands as RARs. It competes with RARs for RXRs, preventing the heterodimerization of RAR/RXR, and thereby inhibiting the function of RAR (DiRenzo et al., 1997).

It has been reported that RARs can participate in mediating the cardiac looping defects in zebrafish induced by the TPhP, an effective agonist of PPARγ (Belcher et al., 2014). To validate the hypothesis that TPhP interferes with the PPARγ and RAR-mediated signaling pathways leading to cardiac developmental toxicity in zebrafish, Mitchell et al. (2018) subjected zebrafish embryos to TPhP for acute exposure, there was a disruption in cardiac development, with impacts on the signaling pathways of five RXR-related nuclear receptors. The expression of TCAP, fatty acid-binding protein 1b, and desmin a in the myocardium was upregulated, indicating that TPhP induces cardiotoxicity through the nuclear receptor pathway. However, TPhP may exert its effects by indirectly influencing the binding of upstream RXRs, rather than binding directly to RXRs. At present, it remains uncertain whether TPhP can function as a ligand for RXRs, and its mode of action is still not well-defined. Further investigation could be carried out by knocking out RXRs to gain deeper insights into this matter. Moreover, 15 ligands were selected that can alleviate pericardial edema (Mitchell et al., 2018). Among them, fenretinide (RARs agonist) and ciglitazone (PPARγ agonist) reduced (in a concentration-dependent-manner) the cardiotoxicity caused by TPhP, indicating that these two agonists can be used as drugs to prevent or treat this toxic effect.

Other studies have shown that the cardiotoxicity induced by mITP also involves the nuclear receptor signaling pathways. The mITP and TPhP are the main components in the composite additive organic phosphorus flame retardant Firemaster 550 (FM 550). They both belong to aryl OPEs and have similar structures, with the difference being the presence of isopropyl groups in mITP. The mITP can downregulate *cyp26a1*, *dhr3a,* and *dhrs3b*, and may play a role by inhibiting RAR (Haggard et al., 2017). The Cyp26a1 enzyme is responsible for the metabolic degradation of the RA, whereas *Dhr3a* and *Dhrs3b* are responsible for the degradation of all-trans retinol to vitamin A (Feng et al., 2010). The *Hox* gene family is involved in regulating the fate of heart cells in SHF (Waxman et al., 2008), and its expression is regulated by RA signaling (Ahn et al., 2014). The results showed that the expression of the *Hox* gene family (*hoxb5b*, *hoxb6b*, *hoxa5a*, *hoxc1a*, and *hoxb8b*) was significantly reduced, indicating that mITP inhibition of RARs led to downregulation of the *Hox* gene expression and the occurrence of cardiac abnormalities. We speculate that the effective small molecule modulators fenretinide and ciglitazone for TPhP are still effective in treating mITP-induced cardiac toxicity, which requires further confirmation.

### 3.4 Aromatic hydrocarbon receptor pathway

The aromatic hydrocarbon receptor (AHR), as a member of the basic helix-loop-helix transcription factor family, functions as a sensor in organisms for responding to external environmental stimuli. Upon ligand activation, the AHR translocates from the cytoplasm into the nucleus, where it forms a dimer with an AHR nuclear translocator. Subsequently, it regulates gene transcription, activating downstream signaling pathways associated with cellular toxicity. The target genes include cytochrome P450 superfamily members, reduced nicotinamide adenine dinucleotide phosphate, quinone oxidoreductase, and aldehyde dehydrogenase (Zhang, 2011). The AHR is involved in the initiation and progression of the cardiovascular system and its associated diseases. The *AHR* gene knockout mice exhibit myocardial hypertrophy, vascular remodeling, and systemic hypertension (Lund et al., 2003; Lund et al., 2008). Conversely, excessive activation of AHR can mediate inflammatory responses, leading to atherosclerosis in mice (Wu et al., 2011). It has been found that 2,3,7,8-tetrachlorodibenzo-p-dioxin (TCDD) can inhibit epicardial formation during cardiac development by activating the AHR pathway, resulting in developmental malformations, volume reduction, decreased cardiomyocyte numbers, blood regurgitation, and conduction block in the heart (Antkiewicz et al., 2005; Hofsteen et al., 2013). TCDD-like compounds and polycyclic aromatic hydrocarbons (PAHs) are the two main types of environmental pollutants known to activate the AHR signaling pathway. Therefore, AHR is also referred to as the “dioxin receptor” due to its responsiveness to these compounds.

The mITP is another ligand of the AHR. Its effects on cardiac looping and pericardium area in zebrafish embryos can be mitigated by co-exposure with an AHR antagonist CH223191, but no significant effect on heart rate has been found compared with mITP alone treatment (McGee et al., 2013). Further studies using a functional zebrafish AHR2 knockout line along with AHR1A- and AHR1B-specific morpholinos revealed that mITP interacted with both AHR2 and AHR1B and induced the expression of cytochrome P450 1A, but knockout all three AHR subtypes did not block the mITP-induced cardiotoxicity (Gerlach et al., 2014). In addition, similarly to TPhP, mITP can also cause pericardial oedema, abnormal cardiac cyclisation, and reduced heart rate in zebrafish embryos through the RARs signaling pathway (Haggard et al., 2017). The above results suggest that the cardiotoxicity of mITP in zebrafish can be mediated through RARs and AHR signaling pathways.

**FIGURE 1:**
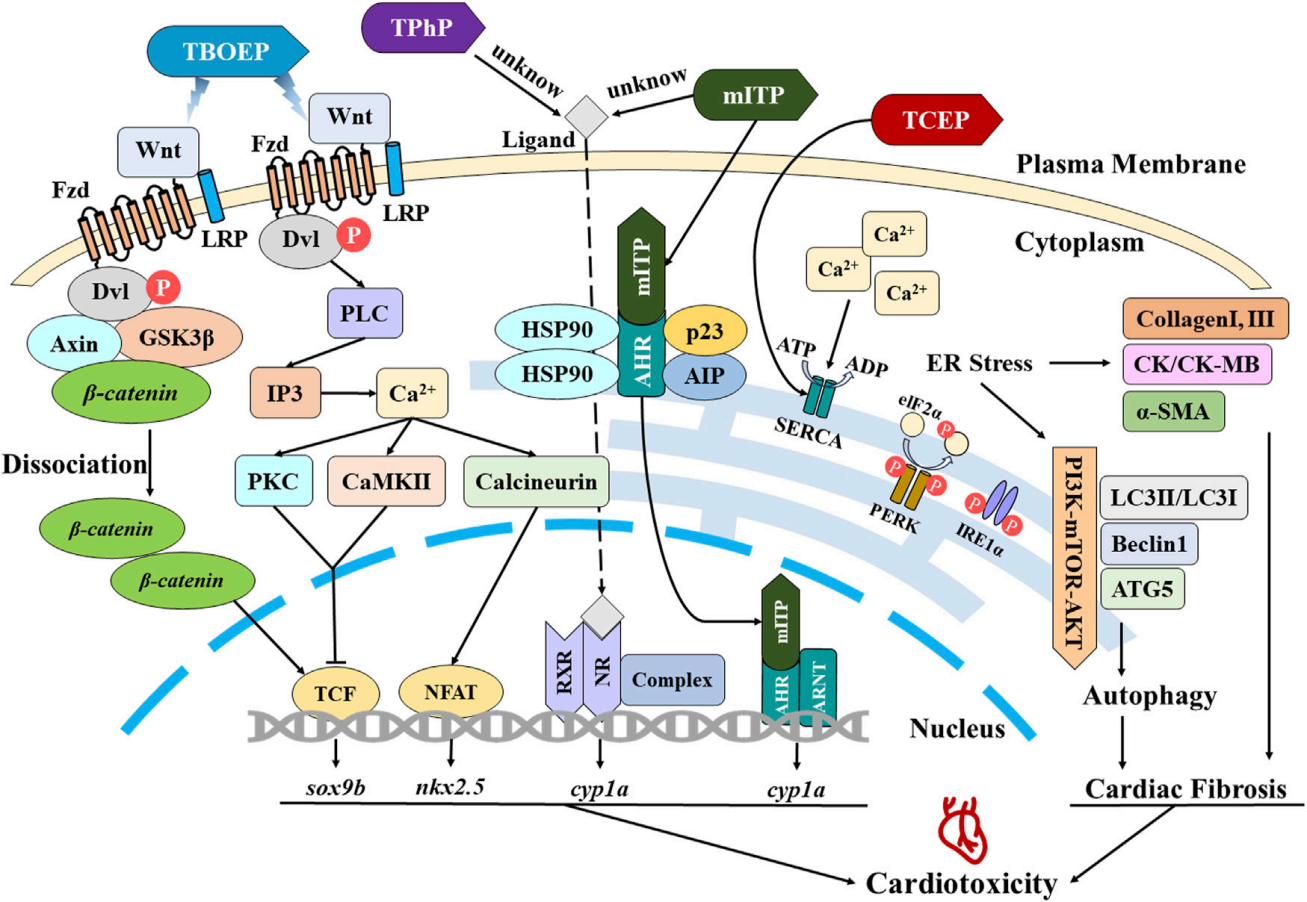
Schematic representation of mechanisms associated with cardiotoxicity induced by OPEs.

The pertinent cellular and molecular mechanisms underlying cardiotoxicity induced by OPEs have been summarized, and a schematic diagram (Figure 1) is constructed to illustrate the intracellular signaling pathways.

## 4 Conclusion and outlook

As the latest generation of flame retardants, the extensive utilization of OPEs in industrial and agricultural production has presented a potential environmental and biological health hazard. Numerous studies have substantiated that OPEs exhibit high environmental persistence, are readily absorbed and accumulated by living organisms, and can induce diverse toxic effects including neurotoxicity, metabolic toxicity, reproductive toxicity, cardiotoxicity, and immune toxicity. Currently, investigations on the cardiotoxicity of OPEs have primarily been conducted in zebrafish, along with other fish species, birds, mammals, and *in vitro* cells. Generally, the exposure to OPEs can elicit cardiac morphological and structural alterations such as pericardial edema and abnormal cardiac cyclization (e.g., prolonged SV-BA distance or dioxin-like tubular heart), decreased number of myocardial cells, thinning of atrioventricular walls, myocardial fibrosis and hypertrophy, as well as physiological functional changes such as decreased heart rate, bradycardia, weakened cardiomyocyte pulsation ability, and mitochondrial metabolic disorder. Additionally, the exposure to OPEs can induce alterations in the expression levels of key molecules and biomarkers, including *bmp4*, *nkx2.5*, *gata4*, *tbx5*, *Hox* gene family, CK, CK-MB, TCAP, collagen type I, collagen type III, and *α*-SMA, etc. These aforementioned effects may be mediated through pathways like the Wnt signaling pathway, Ca^2+^ overload/ER stress/autophagy axis, nuclear receptor or AHR pathways.

However, there might be variations in mechanisms of toxicity and manifestations between different types of OPEs. For instance, TBOEP (alkyl OPEs) has been found to induce developmental toxicity on the heart of zebrafish larvae through the Wnt signaling pathway. TCEP (chlorinated OPEs) can trigger ER stress in cardiomyocytes, leading to calcium overload in the ER and disrupting mitochondrial structure, ultimately initiating autophagy. TPhP (aryl OPEs) has been shown to have adverse effects on cardiac differentiation and development through the nuclear receptor pathway, however it remains uncertain whether it acts directly or indirectly upon this pathway. Aryl OPEs have demonstrated a higher potency in inducing cardiotoxicity compared to the other two types. Furthermore, within the same type of OPEs, their pathways may be different. For example, both TPhP and mITP can induce cardiotoxicity in zebrafish through the RARs signaling pathway; however, mITP can also be mediated by the AHR pathway.

The current studies primarily focus on heart rate as the indicators of the effects of OPEs on cardiac physiological function. However, future research should also consider incorporating commonly used measures such as cardiac output and ejection fraction to comprehensively evaluate cardiac function. For instance, the administration of various targeted therapies for metastatic NSCLC may result in QT interval prolongation, supraventricular tachycardia or ventricular arrhythmias and potentially even heart failure (Waliany et al., 2021). The compound enzastaurin possesses the capability to inhibit potassium channels in myocardial cells of guinea pig (*Cavia porcellus*), resulting in an increase in action potential duration and prolongation of the QT interval, ultimately inducing a negative chronotropic effect (Zhang et al., 2023). The current utilization of this technique in the investigation of cardiotoxic effects caused by environmental pollutants (e.g., OPEs, etc.) on organisms is limited. However, its future implementation could be considered to enhance the comprehensiveness of assessing the environmental toxicological effects of pollutants.

Most studies report a variety of toxic effects of OPEs on organisms, there are also studies indicating that TDCPP, at low exposure concentrations, can attenuate hydrogen peroxide-induced Ca^2+^ overload in H9C2 cells by reducing Ca^2+^ inward flow, reduce excessive autophagy, and mitigates myocardial oxidative stress injury by activating the PI3K/Akt/GSK3β signaling pathway (Zhang et al., 2019). This offers novel insights for rational applications of OPEs.
